# Rare “T-junction” aberrancy in vascular anatomy of the pectoral branch of the thoracoacromial trunk in the pectoralis major myocutaneous flap: A case report

**DOI:** 10.1016/j.jpra.2025.06.010

**Published:** 2025-06-23

**Authors:** B. Sio, SL. Zhang, HW Ng.

**Affiliations:** a Plastic, Reconstructive and Aesthetic Surgery Service, Department of General Surgery, Tan Tock Seng Hospital, Singapore, Singapore; b Plastic, Reconstructive and Aesthetic Surgery Service, Department of Surgery, Woodlands Health, Singapore, Singapore

**Keywords:** Pectoralis major myocutaneous flap, Pectoral branch of thoracoacromial trunk, Aberrancy, Head and neck reconstruction

## Abstract

*Study Design: Case Report:* The Pectoralis Major Myocutaneous Flap (PMMF) is easily harvested for Head and Neck reconstruction due to its consistent vascular anatomy and reliability of its main axial pedicle – Pectoral Branch of the Thoracoacromial Trunk (PB-TAT). Although the anatomical course of the PB-TAT is extensively studied, giving reconstructive surgeons the knowledge and confidence of utilising the PMMF in their armamentarium, we describe a case of its aberrancy which led to an unpremeditated ligation of this main pedicle, resulting in on-table flap failure. Instead of the expected submuscular course of the PB-TAT from the mid-clavicular line to join the acromio-xiphoid axis as it courses medially in a cranio-caudal fashion, we identified this pedicle in our case to divide into its medial and lateral branches in a 180 degrees “T-junction” fashion as it approaches the acromio-xiphoid axis, and entering the pectoralis major muscle only after a short extra muscular course. Despite harvesting the lateral extent of the PMMF further lateral to where the lateral branch of the PB-TAT entered the muscle, majority of the flap was not bleeding after elevation, suggesting an inadvertent ligation of this main pedicle. Although the vascular anatomy of the PMMF has been extensively studied in the literature, we believe this aberrancy in the course of the PB-TAT has not been described, and hope to share this encounter with other reconstructive surgeons, to be vigilant and mindful even when raising a ‘straight-forward’ flap.

## Introduction

The Pectoralis Major Myocutaneous Flap (PMMF) has been a workhorse flap for Head and Neck reconstruction due to its reliable vascular anatomy and ease of harvest. The Pectoralis Major (PM) is a Mathes and Nahai type-V muscle flap.^1^ with its main vascular pedicle being the Pectoral Branch of the Thoracoacromial Trunk (PB-TAT). Contributory supplies also come from the pectoral branch of the lateral thoracic artery and segmental perforating branches of the internal mammary artery*.*^2^, ^3^, ^4^

**Figure 1:**
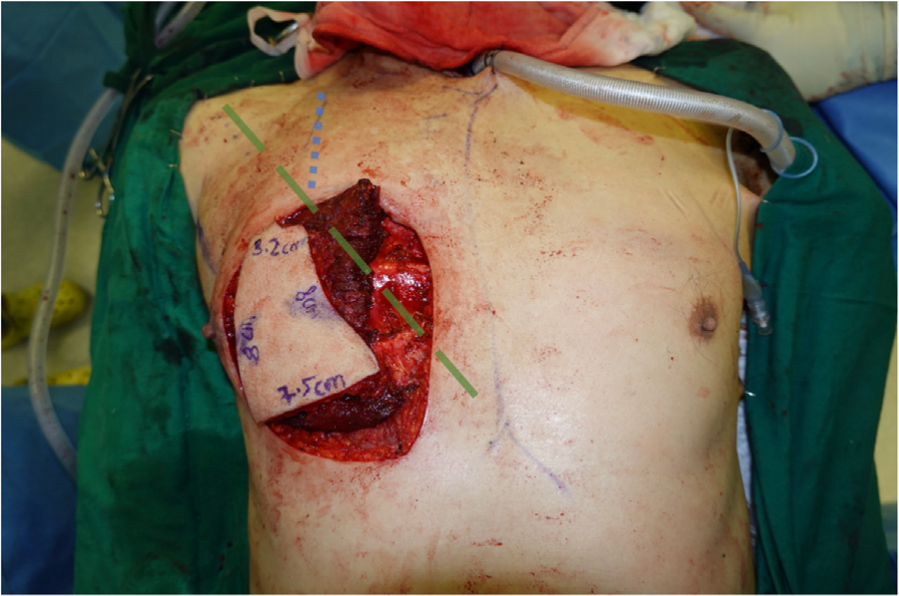
Mid-clavicular line (blue dotted line) and acromio-xiphoid axis (dashed green line) superimposed. Skin paddle designed entirely over PM muscle, raised off anticipated course of PB-TAT as described by Ariyan 1979. Medial extent of dissection at sternal border, as seen with exposed costal cartilages.

The flap (sternocostal segment of PM) can be raised completely based off the PB-TAT as the axial vessel,^5^, ^6^, ^7^, ^8^ in a pedicled manner or as a free flap. The technique of pedicled-PMMF elevation was first described by Ariyan in 1979,^9^ and the surgical principle is still relevant today. Ariyan described the pedicle axis of the PB-TAT to follow a vertical course down from the mid-clavicular point, joining a line from the acromion to xiphoid as it travels medially (Figure 1). The PB-TAT deviates from this acromio-xiphoid axis for <30 degrees in most cases.^3^^,^^7^

The well-understood and predictable vascular anatomy, ease of elevation, and ability to directly visualize its vascular pedicle to prevent inadvertent injury has made the PMMF a flap with low failure rates and a popular reconstructive option for the reconstructive surgeon, particularly in cases of salvage in Head and Neck reconstruction.

The authors describe a rare case of vascular aberrancy of the PB-TAT, which resulted in unpremeditated ligation of this pedicle, and on-table flap failure.

To our knowledge, such a vascular anatomy of the PM muscle has not been described before. We aim to share this encounter with other reconstructive surgeons, to be vigilant and mindful for any anatomical aberrancy, when raising a supposedly ‘straight-forward’ pedicled flap.

## Case report

Our patient is a 65 year old male with recurrent right glottic Squamous Cell Carcinoma after radiation therapy, and underwent total laryngectomy and bilateral neck dissection. Pedicled-PMMF reconstruction for the anterior pharyngeal wall defect was planned. His past medical history included hypertension, hyperlipidaemia, diabetes-mellitus and May-Thurner Syndrome with provoked left iliofemoral deep vein thrombosis.

Preoperative design of the pedicled-PMMF was done in the usual fashion, marking the anticipated course of the PB-TAT as described by Ariyan 1979 (Figure 1).^9^ The skin paddle was planned entirely over the PM muscle along the inferior edge.

### Surgical conduct

**Figure 2:**
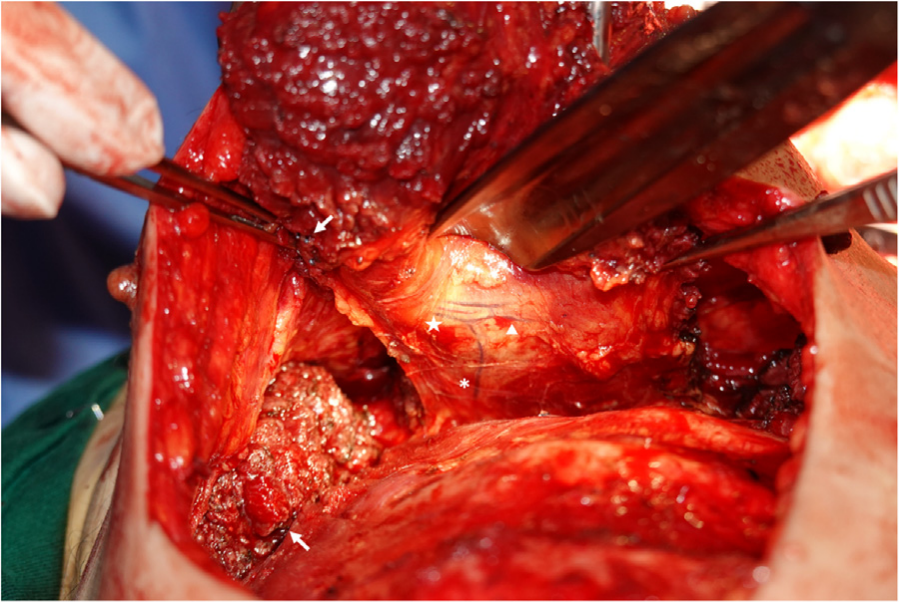
Aberrancy of PB-TAT splitting into lateral and medial branches in a 180 degrees fashion (“T-Junction”) and entering the muscle after a short sub-pectoral course. Asterix, PB-TAT; Star, lateral branch of PB-TAT; Triangle, medial branch of PB-TAT; Arrows, clips where pectoral branch of lateral thoracic artery was ligated.
○ The lateral extent of flap harvested was taken at a distance lateral to where the lateral division of the PB-TAT entered the muscle (Figure 3).

○ After incision along the skin paddle, the PM muscle was identified and raised caudo-cranially in the avascular sub-pectoral plane.
○ The PM muscle was released at the sternal border medially.
○ At the lateral border, the pectoral branch of the lateral thoracic artery was identified and ligated to allow greater reach during transposition of the flap to the neck.
○ The PB-TAT was identified coursing under the surface of the PM muscle within the fascial envelope, parallel and just lateral to the mid-clavicular line, travelling approximately 6–7 cm before dividing into medial and lateral branches at a 180-degree angle (“T-junction” fashion) and entering the muscle substance after a short course (Figure 2).

**Figure 3:**
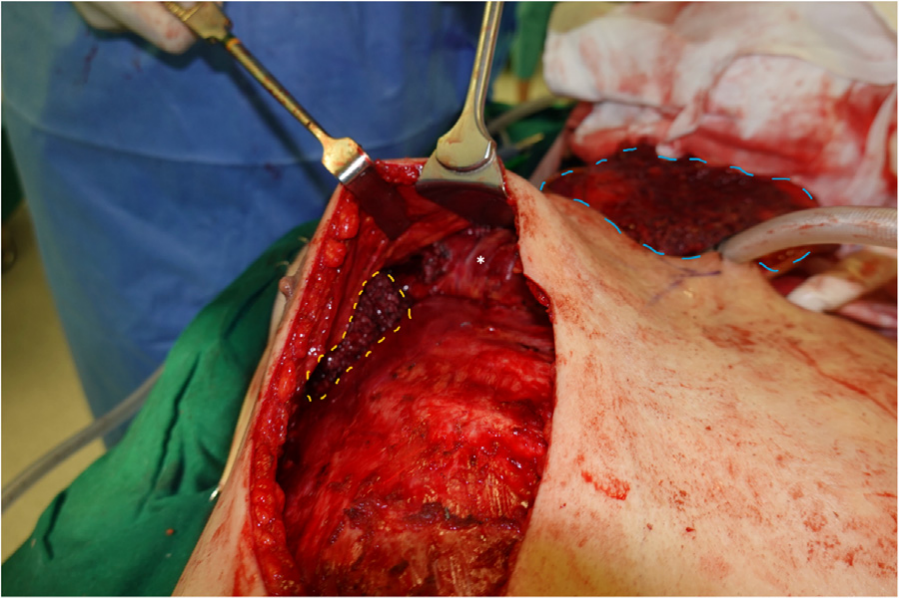
Lateral extent of dissection in elevation of PMMF (broken yellow line), with tunnelled PMMF to neck (broken blue line). Asterix, PB-TAT.
○ Elevation of the flap was taken up till the clavicle attachment to allow sufficient reach to the neck, encompassing and protecting the PB-TAT until its base.

After flap raising, the skin paddle and muscle was noted not to be bleeding. Intraoperative indocyanine green angiography confirmed complete absence of perfusion of the flap distal to the “T-junction” where the PB-TAT diverged into its medial and lateral branches at 180-degrees. 80 % of the flap was debrided as it was ischaemic and bleeding was only encountered at the level of division of the PB-TAT. Following discussion with the resecting surgeon, the flap was abandoned and the anterior pharyngeal wall defect was closed primarily, with a view for staged reconstruction with a contralateral PMMF should the patient develop a salivary leak or stricture later on.

## Discussion

Anatomical studies of over 70 PM muscles by Park HD et al.^10^ had demonstrated that the course of the PB-TAT lies within 2 cm from the mid-clavicular line in majority of the dissection, as in this case. In the examination of 109 vessels, Friedrich W et al.^7^ described that the greatest deviation in the course of the PB-TAT from the reference line of the acromio-xiphoid axis was only at 34 degrees. Additionally, when the PB-TAT splits to >1 branch, the dividing angle is only up to 30 degrees. Similarly, Ducasse A et al.^3^ demonstrated the deviation in the course of the PB-TAT from the acromio-xiphoid axis to be <30 degrees in majority of cases.

Our case presents a unique situation where the PB-TAT splits into its medial and lateral branches in a 180-degree horizontal “T-junction” configuration after traversing along the mid-clavicular line, significantly deviating from the commonly described path along the acromio-xiphoid axis. These branches took a short submuscular course before entering the PM muscle substance, and supplying the rest of the sternocostal portion of the PMMF.

To the authors’ knowledge, such vascular aberrancy of the PB-TAT has not been observed before or described. Despite detaching at the sternal border and taking the lateral border of the PMMF at some distance after the lateral division of the PB-TAT entered the muscle, it was evidently inadequate to encompass the flap’s vascular supply from the pedicle, resulting in flap failure.

**Figure 4:**
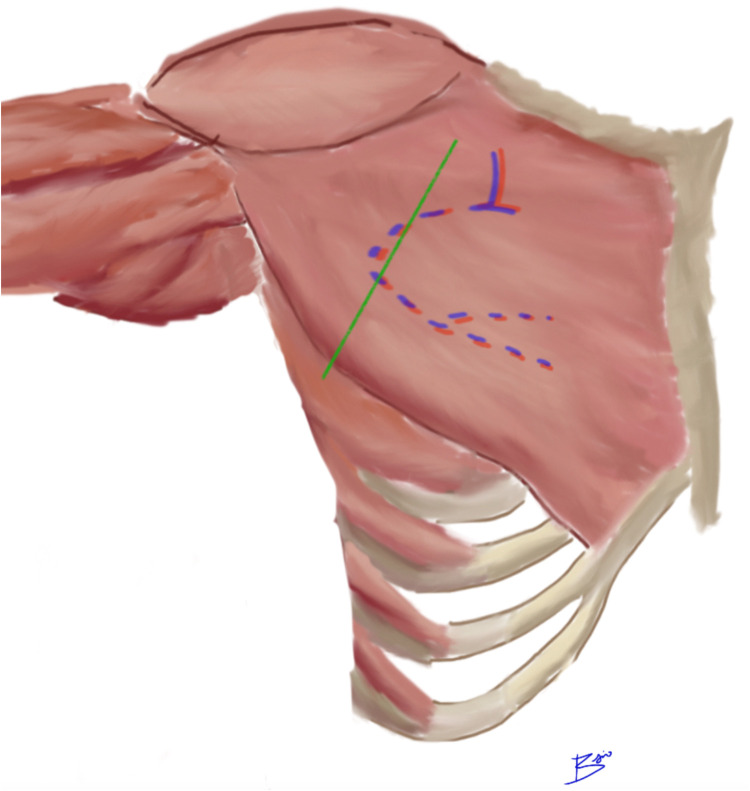
Schematic illustration of course of PB-TAT dividing 180-degrees to medial and lateral branches (“T-junction” fashion). Dotted red and blue lines represent postulated intramuscular course of lateral branch of artery and vein respectively. Green line represents lateral extent of flap transection.

The author’s postulate that the lateral division of the PB-TAT had travelled further laterally in its intramuscular course, before making an acute turn and arborizing to supply the distal and perhaps more medial segments of the muscle (Figure 4). As a result, the vascular supply to the flap was transacted while dividing the lateral border.

Based off previous studies where the PMMF can be raised purely on the PB-TAT,^5^, ^6^, ^7^, ^8^ in our case of vascular aberrancy of the PB-TAT, the authors suggest the safest technique for flap elevation is to elevate the PM muscle as far laterally as close to the humeral insertion, before cutting into the muscle to determine the lateral extent of the flap and narrowing it at its pedicle. This will ensure a broad coverage of the possible pedicle’s intramuscular course, although leading to a larger/bulkier flap.

It is not routine for pre-operative CT-angiography to delineate the normally reliable vascular anatomy of the PMMF. However the authors suggest the use of intraoperative doppler ultrasound may be beneficial if aberrant branching of the PB-TAT is identified, which may assist in delineating its intramuscular course.

Incidentally, the patient also has May-Thurner Syndrome, a vascular anomaly characterised by compression of the left iliac vein by the right iliac artery which courses over it. Further studies are required to investigate if this syndrome is indeed associated with a predisposition to/increased risk of vascular aberrancy in other parts of the body (including the PB-TAT).

Precise knowledge of the vascular supply to the flap is key in successful flap elevation. Despite extensive studies on the vascular anatomy of the PMMF since its description in the 1980s, aberrancy that has not yet been described may still be encountered. We hope this case reminds reconstructive surgeons to be vigilant during flap dissection and adopt precautionary measures in elevating a flap that may be relatively “straight-forward”.

## Ethical approval

Not required.

## Funding

None.

## Declaration of competing interest

The authors declare that they have no known competing financial interests or personal relationships that could have appeared to influence the work reported in this paper.
